# Circulating PGRN Levels Are Increased but Not Associated with Insulin Sensitivity or *β*-Cell Function in Chinese Obese Children

**DOI:** 10.1155/2018/3729402

**Published:** 2018-07-29

**Authors:** Fengyun Wang, Ting Chen, Ling Sun, Haitao Lv, Xuan Li, Jie Shen, Linqi Chen, Zhenyu Chu, Miao Hou

**Affiliations:** ^1^Department of Endocrinology, Metabolism and Genetic Disorders, Children's Hospital of Soochow University, Suzhou, Jiangsu, China; ^2^Department of Cardiology, Children's Hospital of Soochow University, Suzhou, Jiangsu, China; ^3^Department of Emergency Intensive Care Unit, The First Affiliated Hospital of Soochow University, Jiangsu, China

## Abstract

Progranulin (PGRN), a novel peptide that has recently emerged as an important regulatory adipokine, is relevant to energy homeostasis and obesity in animals and adult humans. Little is known about its roles in children. The aim of the current study was to determine the potential role of PGRN and explore its relationship to various obesity-related markers in obese children. This was a cross-sectional study composed of 77 children (43 obese and 34 healthy, age 8.68 ± 0.28 and 8.46 ± 0.45 years, resp.). The PGRN levels were significantly higher in obese children (102.44 ± 4.18 ng/mL) comparing to controls (69.32 ± 5.49 ng/mL) (*P* < 0.05). Moreover, the PGRN levels were positively correlated with triglyceride (TG), total cholesterol (TC), IL-6, systolic blood pressure (SBP), and diastolic blood pressure (DBP) in obese children after adjusted for BMI and age. However, there was no correlation of serum PGRN levels with OGTT-derived dynamic parameters, HOMA-IR, or HOMA-*β* in obese children. The results suggest that serum PGRN levels are significantly higher in obese children in China and correlate significantly with obesity-related markers. Increased PGRN levels may be involved in the pathological mechanism of childhood obesity.

## 1. Introduction

Childhood obesity has become a global public health issue. The prevalence of obesity has tripled in the last three decades. Among Chinese children, the combined prevalence of overweight and obesity has increased rapidly over the past decades, from less than 3% in 1985 to 19.2% in 2010 [1]. Childhood obesity is associated with a number of adverse health consequences including type 2 diabetes (T2DM), dyslipidemia, and hypertension, all of which will lead to premature cardiovascular diseases [2, 3].

As is well known, obesity is defined as excess fat mass accumulation. Adipose tissue, in addition to energy storage, has been found to have a variety of endocrine functions. It can secrete all kinds of adipokines [4, 5], including leptin, adiponectin, and resistin, all of which play important roles in metabolism and energy homeostasis. Progranulin (PGRN), also known as proepithelin, is a pluripotent growth factor that mediates cell growth, wound healing, tumorigenesis, and neurodegenerative disease [6, 7]. More recently, PGRN has emerged as an important regulatory adipokine of glucose metabolism and insulin sensitivity [8, 9]. For instance, diet-induced obese mice with PGRN deficiency exhibited lower body weight and ameliorated insulin sensitivity, whereas administration of recombinant PGRN induced obesity and glucose intolerance in wild-type mice with standard diet [10]. Consistently, PGRN affects insulin signaling and suppresses insulin-stimulated glucose uptake in 3T3-L1 adipocytes [10]. Moreover, several clinical investigations also demonstrated that serum PGRN was associated with parameters of adiposity, glucose tolerance, and inflammatory factors [11, 12]. In patients with T2DM, circulating PGRN is significantly higher comparing to normal controls and positively correlates with high-sensitivity C-reactive protein, IL-6, and macrophage infiltration in omental adipose tissue (AT) [13]. In particular, PGRN expression in visceral AT is higher than in subcutaneous AT of insulin-resistant patients [14].

Up to now, the clinical data have revealed a relationship between PGRN levels and obesity. However, few studies have explored the PGRN levels in obese children. Therefore, the purpose of the present study was to investigate possible correlations between PGRN levels and obesity in Chinese children, and to identify associations between PGRN levels and obesity-related disorders.

## 2. Materials and Methods

### 2.1. Study Design

The study was initiated upon approval of the local ethics committee of the Faculty of Medicine of Soochow University, in light of the Helsinki Declaration. A written informed consent of the parent(s) of each subject was obtained before the study.

This study recruited 43 obese children, 13 girls and 30 boys, with BMI above the 95th percentile. Another 34 healthy subjects with BMI below the 85th percentile with similar age and gender distribution were enrolled as controls.

Before the outset of the study, all the patients and control subjects had under taken general physical examination and laboratory evaluation to exclude other illnesses. Those with chronic diseases (cardiovascular, gastrointestinal, or respiratory), history of drug use (steroids or antipsychotics), endocrine disorders (Cushing syndrome or hypothyroidism), or suspected syndromes associated with obesity (Prader-Willi or Laurence-Moon-Biedl syndromes) were excluded from the study. Pubertal development of subjects was evaluated according to Tanner staging [15]. Boys with testicular volume larger than 4 mL and girls with breast development more than Tanner stage II were also excluded to avoid the effect of sex hormones on obesity and relative parameters.

### 2.2. Assays and Calculations

Height was measured in the standing position, without shoes, using a stadiometer (sensitivity, 0.1 cm), and weight was measured using a portable scale (sensitivity, 0.1 kg) with the patients dressed in light clothing. BMI was calculated by dividing weight (kg) by squared height (m^2^).

Systolic blood pressure (SBP) and diastolic blood pressure (DBP) were measured twice at the right arm after a 10 min rest in the supine position using a calibrated sphygmomanometer. For oral glucose tolerance test (OGTT), a 180 min OGTT (1.75 g/kg glucose, maximum 75 g) was performed in the morning after 10 to 12 hours overnight fasting. Blood samples were obtained by an antecubital venous catheter at 0, 30, 60, 120, and 180 min for determination of glucose, insulin, and C-peptide levels as described previously [16].

The homeostasis model assessment for insulin resistance (HOMA-IR) was calculated using the following formula: HOMA-IR = Insulin (mU/mL) × Glucose (mmol/L)/22.5, as previously described [17]. HOMA-IR > 2.5 was used as a cutoff value to differentiate insulin resistant from nonresistant obese subjects [18].

To assess the *β*-cell function, the homeostasis model assessment for *β*-cell function (HOMA-*β*) was calculated as follows: 20 × Insulin (mU/mL)/[Glucose (mmol/L)-3.5] [19]. Moreover, the insulinogenic index and comparable C-peptide index (*Δ*I30/ΔG30, ΔC30/ΔG30) were calculated as the ratio of the incremental change of insulin or C-peptide to glucose, respectively, from 0 to 30 min of the OGTT as previously reported [20].

### 2.3. Laboratory Analysis

Blood samples for glucose, insulin, lipid profiles, and PGRN levels were taken after 10–12 h night fasting. Blood was obtained from an antecubital venous catheter and placed on ice. Serum was separated within 20 min and stored at −80°C until analysis.

Fasting glucose was assayed by glucose oxidase method. HbAC1 was measured by isoelectric focusing. Serum insulin levels were measured by RIA using human insulin as standard (Millipore, Catalog number: EZHIASF-14K). Triglyceride (TG), total cholesterol (TC), high-density lipoprotein cholesterol (HDL), low-density lipoprotein cholesterol (LDL), liver function, and high-sensitive CRP concentrations were detected by autoanalyzer (Beckman CX-7 Biochemical Autoanalyser, Brea, CA, USA).

Serum PGRN, IL-6, and TNF*α* analysis was all performed with the ELISA kits. PGRN concentrations were determined with ELISA kit (R&D, Catalog number: DPGRN0). The ELISA kit has a dynamic range between 1.6 and 100 ng/mL and a detection limit of 0.54 ng/mL. Intra-assay and interassay coefficient of variations (CV) were <4.4% and 7.4%, respectively. IL-6 and TNF*α* concentrations were determined with ELISA Kit (eBioscience company, Catalog number: BMS213-2TEN and BMS2034, resp.). The IL-6 ELISA kit has a dynamic range between 15 pg/mL and 1540 pg/mL and a detection limit of 2 pg/mL. Intra-assay and interassay CVs were 5.6% and 7.5%, respectively. The assay range for TNF*α* ELISA kit was 7.8–500 pg/mL, sensitivity was 2.3 pg/mL, and intra-assay and interassay CVs were 6.0% and 7.4%, respectively.

### 2.4. Statistical Analyses

Statistical analyses of the data were conducted by SPSS 19.0.1 (SPSS Inc., Chicago, IL, USA). All values are presented as mean ± S.E.M. Distribution of data was evaluated with the Kolmogorov-Smirnov test. For numerical comparisons, Student's *t*-test (between obese and control groups and for insulin-resistant and nonresistant subgroups) was used. Categorical variables were compared using chi-squared test. The correlation between the PGRN levels with demographics and clinical characteristics was investigated with Pearson's correlation analysis and partial correlation analysis after adjusting for age and BMI. *P* < 0.05 was considered statistically significant.

## 3. Results

### 3.1. The Clinical Characteristics

A total of 77 subjects were enrolled in this study, including 43 subjects (13 girls and 30 boys) in an obesity group and 34 subjects (12 girls and 22 boys) in a control group. Average age was 8.68 ± 0.28 years and 8.46 ± 0.45 years in the obesity group and control group, respectively.  Table 1 summarizes the demographics and clinical characteristics of both groups. Among all subjects, those in the obesity group had significantly higher BMI, SBP, DBP, TG, LDL-C, insulin, HbAC1, GPT, HOMA-IR, HOMA-*β*, insulinogenic index, C-peptide index, hsCRP, IL-6, and TNF-*α* (all *P* < 0.05) levels than the control group, whereas HDL-C levels were lower in obesity group compared with control group. Moreover, the levels of insulin, HOMA-*β*, and insulinogenic index were higher in insulin-resistant obese subjects compared to noninsulin-resistant obese subjects (Table 2).

### 3.2. The Changes in Serum PGRN Concentrations in Obese Children

**Figure 1:**
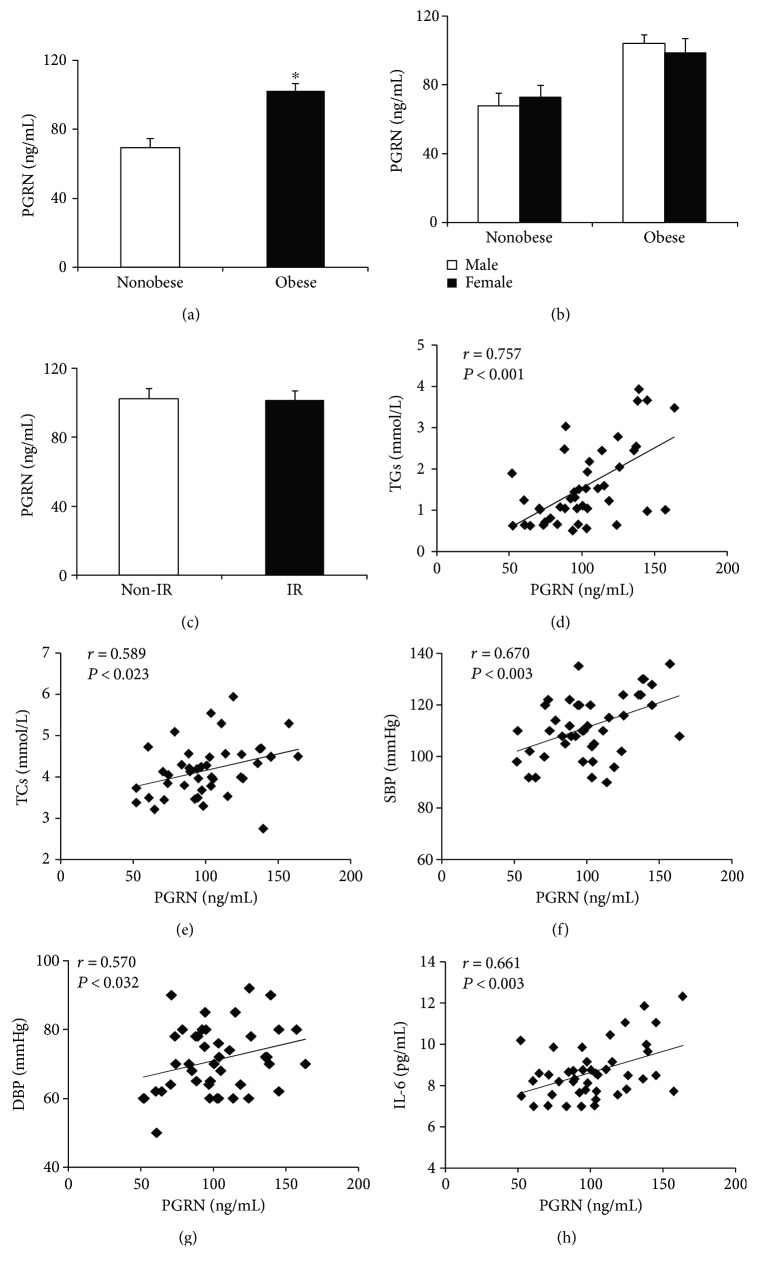
Serum PGRN levels and its correlation with clinical characteristics. (a) PGRN serum levels in nonobese and obese children, (b) PGRN serum levels between boys and girls in nonobese and obese group, (c) PGRN serum levels in noninsulin-resistant (non-IR) obese subject and insulin-resistant (IR) obese subject, and (d–h) scatter plots showing the correlation of serum PGRN levels with TG (d), TC (e), SBP (f), DBP (g), and IL-6 (h) in obese subjects. Data are expressed as mean ± SEM. ^∗^*P* < 0.05 for nonobese versus obese children.

Compared to control group, obesity group displayed a significant increase in the PGRN concentrations (Figure 1(a)). However, there were no significant differences in serum PGRN concentrations between boys and girls (Figure 1(b)), either in the obesity group or in the control group. Moreover, no significant differences were detected in serum PGRN levels between the noninsulin-resistant obese subjects (*n* = 26) and the insulin-resistant obese subjects (*n* = 17) (Figure 1(c)).

### 3.3. Association of Serum PGRN Concentrations with Metabolic Parameters

In obese subjects, the serum PGRN concentrations correlated positively and significantly with BMI, TG, TCs, SBP, DBP, and IL-6 (Table 3) levels. After adjusting for age and BMI, PGRN still correlated positively and significantly with TGs, TCs, SBP, DBP, and IL-6, respectively (Figure 1(d)–1(h)). However, there were no correlations between serum PGRN levels and insulinogenic index, HOMA-IR, or HOMA-*β* in obese children.

## 4. Discussion

Progranulin is a 68–88 kDa multifunctional protein, which was originally discovered by Anakwe and Gerton in 1990 [21], and has been implicated in cell growth, wound repair, tumor genesis, neurodevelopment, neurodegeneration, and more recently, energy metabolism regulation [8, 9]. The present study analyzed the data of obese Chinese children, aiming to investigate whether correlations could be found between PGRN levels and obesity in this population.

To the best of our knowledge, this is the first study on PGRN and obesity-related markers in Chinese children. We found that serum PGRN concentrations were 1.5-fold higher in obese children, comparing to controls with normal weight. We also found that PGRN levels were positively correlated with BMI, TG, and TC in obese children. Our results were consistent with previous studies by Alissa and colleagues, who classified Saudi Arabia children into four groups based on quartiles of serum PGRN levels and found that children within the upper quartile of serum PGRN concentration were heavier and had higher concentrations of serum TC and TGs comparing to those in the lower quartile [22]. Moreover, in the previous study conducted by Qu et al., it was found that circulating PGRN concentrations were higher in obese group than healthy subjects and correlated positively with BMI [8]. Consistently, Li et al. [23] proved that serum PGRN concentrations were significantly higher in patients with metabolic syndrome (MS) than in subjects without MS and correlated positively with BMI and waist circumference. Results of these studies all indicate that increased circulating PGRN concentrations were closely related to measures of obesity, both in adults and children. The reasons behind elevated serum PGRN in obese subjects are still a matter of discussion. Our findings shed some light, implying that enhanced synthesis of this adipokine may result from augmented adipose tissue in obese subject, since adipose tissue matrix expresses PGRN gene [10], and it is the important source for circulating PGRN [24]. To verify this hypothesis, further studies are needed to analyze the expression of PGRN expression in adipose tissue in obese subjects.

We also demonstrated that children presenting elevated levels of IL-6, TNF-*α*, hsCRP, and IL-6 correlated strongly with serum PGRN concentrations. Previous studies in obese adults have also showed a positive correlation between PGRN and inflammatory markers, especially hsCRP [13] and IL-6 [8]. However, it is unclear if the increasing serum PGRN is a consequence of obesity-associated inflammation, or rather the latter is triggered due to the overproduction of PGRN. Nevertheless, the fact that elevated serum concentrations of PGRN were also previously observed in other chronic inflammation diseases, such as asthma [25], systemic lupus erythematosus [26], arthritis [27], and neurodegenerative disease [28], suggests this adipokine as a marker of ongoing inflammation, rather than a triggering factor of it. This hypothesis is also supported by the fact that IL-6 could stimulate PGRN expression in vitro [29]. Thus, it can be speculated that the expression of PGRN in obese children may be stimulated by low-grade inflammation caused by obesity.

The present study showed that the HOMA-IR was higher in obese children comparing to nonobese children, suggesting that obese children had impaired insulin sensitivity, which is consistent with previous studies [2, 17, 18]. Moreover, the OGTT-derived dynamic parameters (insulinogenic index, *Δ*I30/ΔG30; C-peptide index, ΔC30/ΔG30) and HOMA-*β* in obese children were higher than control groups in the present study, implying that their islet secretion function was still enough to compensate their rising demand for insulin [30]. We failed to find a correlation between PGRN and HOMA-IR in obese children. This result was inconsistent with several previous studies which showed positive correlations between PGRN and HOMA-IR [8, 31]. Moreover, correlations between PGRN and OGTT-derived dynamic parameters (insulinogenic index, *Δ*I30/ΔG30; C-peptide index, ΔC30/ΔG30) were also not found, which suggests that PGRN does not correlate to islet secretion function. This finding is different from a previous study which showed negative correlation between PGRN and HOMA-*β* [8]. This discrepancy may be due to relatively mild obesity and insulin resistance in the subjects of our study, comparing to those subjects with T2DM [8] and metabolic syndrome [23] in previous studies. Above all, PGRN may not be a good indicator of insulin resistance and insulin secretion function in mild obese children.

Few studies in the literature investigated the relationship between blood pressure and PGRN, especially in children. Qu et al. and Xu et al. have reported positive correlation between PGRN and blood pressure in adults [8, 32]. In the present study, children with excess body weight manifested with significantly higher blood pressure levels than the controls. More importantly, serum PGRN levels positively correlated with SBP and DBP levels after adjusted for BMI, which suggested the elevation of PGRN might act as an independent risk factor for hypertension. As is well known, PGRN may induce inflammation, chronic inflammation may alter endothelial function and reduce the arterial stiffness [33], thereby affecting the blood pressure regulation. However, the role of PGRN in etiopathogenesis of hypertension is still not fully understood.

One potential limitation of this study stems from a relatively small sample size. Furthermore, owing to lack of biological materials, we could not determine the expression of PGRN gene in adipose tissue of the study subjects.

In conclusion, this study showed that serum levels of PGRN were elevated in obese children, and may serve as a marker of ongoing obesity-related inflammation. Furthermore, our study also suggested that the elevation of PGRN levels in obese children may be an early marker and a potential therapeutic target for management of obesity-related disorders.

## Figures and Tables

**Table 1:** The clinical and laboratory characteristics of obese and nonobese groups.

Variable	Obese subject	Nonobese subject	*P* value
Age(years)	8.68 ± 0.28	8.46 ± 0.45	N.S
Boys/girls	13/30	22/12	N.S
BMI (kg/m^2^)	25.85 ± 0.38	15.40 ± 0.20	<0.01
SBP (mmHg)	111.63 ± 1.84	97.15 ± 0.81	<0.01
DBP (mmHg)	71.14 ± 1.48	65.76 ± 1.45	<0.01
Glucose (mmol/L)	4.39 ± 0.09	4.56 ± 0.13	N.S
HbAC1 (%)	4.47 ± 0.07	4.32 ± 0.05	<0.01
Insulin (*μ*U/mL)	14.80 ± 1.28	4.31 ± 0.40	<0.01
HOMA-IR	2.86 ± 0.25	0.88 ± 0.09	<0.01
HOMA-*β*	402.02 ± 53.61	83.12 ± 8.48	<0.01
Insulinogenic index, ΔI30/*Δ*G30 (*μ*U/mL per mmol/L)	42.15 ± 4.56	62.13 ± 4.12	<0.01
C-peptide index, ΔC30/*Δ*G30 (ng/mL per mmol/L)	2.99 ± 1.09	4.05 ± 0.76	<0.01
TG (mmol/L)	1.57 ± 0.15	0.86 ± 0.08	<0.01
TC (mmol/L)	4.17 ± 0.10	4.12 ± 0.13	N.S
LDL (mmol/L)	2.94 ± 0.10	2.70 ± 0.18	<0.05
HDL (mmol/L)	1.3 ± 0.03	1.56 ± 0.05	<0.01
GPT (U/L)	33.37 ± 5.18	17.17 ± 1.62	<0.01
GOT (U/L)	29.88 ± 2.11	26.89 ± 1.47	N.S
IL-6 (pg/mL)	8.79 ± 0.21	7.38 ± 0.18	<0.01
TNF-*α* (ng/L)	15.52 ± 0.56	11.34 ± 1.02	<0.05
hsCRP (mg/dl)	1.89 ± 0.30	0.38 ± 0.08	<0.01

Data are presented as means ± S.E.M. BMI: body mass index; SBP: systolic blood pressure; DBP: diastolic blood pressure; HOMA-IR: homeostasis model assessment of insulin resistance; HDL-C: high-density lipoprotein cholesterol; LDL-C: low-density lipoprotein cholesterol; TC: total cholesterol; TG: triglycerides.

**Table 2:** The clinical and laboratory characteristics of insulin resistant and nonresistant obese subjects.

Variable	IR (*n* = 17)	Non-IR (*n* = 26)	*P* value
Age (years)	8.79 ± 0.45	8.57 ± 0.34	N.S
Boys/girls	11/6	19/7	N.S
BMI (kg/m2)	25.70 ± 0.05	25.94 ± 0.05	N.S
SBP (mmHg)	111.65 ± 3.10	103.73 ± 4.48	N.S
DBP (mmHg)	74.47 ± 2.48	68.96 ± 1.75	N.S
Glucose (mmol/L)	4.39 ± 0.15	4.38 ± 0.12	N.S
HbAC1 (%)	4.59 ± 0.09	4.35 ± 0.09	N.S
Insulin (*μ*U/mL)	23.14 ± 1.48	9.35 ± 0.80	<0.01
HOMA-IR	4.48 ± 0.28	1.79 ± 0.14	<0.01
HOMA-*β*	470.15 ± 70.15	367.95 ± 67.95	<0.01
Insulinogenic index, ΔI30/ΔG30 (*μ*U/mL per mmol/L)	51.74 ± 7.62	34.74 ± 5.15	<0.05
C-peptide index, ΔC30/*Δ*G30 (ng/mL per mmol/L)	2.36 ± 0.27	3.39 ± 1.81	N.S
TG (mmol/L)	1.71 ± 0.27	1.59 ± 0.17	N.S
TC (mmol/L)	4.09 ± 0.18	4.22 ± 0.11	N.S
LDL (mmol/L)	2.82 ± 0.18	3.01 ± 0.11	N.S
HDL (mmol/L)	1.31 ± 0.06	1.30 ± 0.04	N.S
GPT (U/L)	42.89 ± 10.48	27.14 ± 4.98	N.S
GOT (U/L)	34.90 ± 4.36	26.59 ± 1.81	N.S
IL-6 (pg/mL)	8.32 ± 0.18	8.78 ± 0.22	N.S
TNF-*α* (ng/L)	62.82 ± 1.11	60.67 ± 0.05	N.S
hsCRP (mg/dl)	1.82 ± 0.27	1.93 ± 0.48	N.S

Data are presented as means ± S.E.M. IR: insulin resistant; BMI: body mass index; SBP: systolic blood pressure; DBP: diastolic blood pressure; HOMA-IR: homeostasis model assessment of insulin resistance; HDL-C: high-density lipoprotein cholesterol; LDL-C: low-density lipoprotein cholesterol; TC: total cholesterol; TG: triglycerides.

**Table 3:** Correlation of PGRN levels with clinical characteristics in obese groups.

Variable	*r*	*P* value
BMI (kg·m^2^)	0.742	0.001
Age (years)	−0.182	0.850
SBP (mmHg)	0.670	0.003
DBP (mmHg)	0.570	0.032
Insulin (*μ*U/mL)	0.250	0.690
HbAC1 (%)	0.219	0.832
Glucose (mmol/L)	0.080	0.968
HOMA-IR	0.260	0.668
HOMA-*β*	−0.222	0.750
Insulinogenic index, ΔI30/ΔG30 (*μ*U/mL per mmol/L)	0.261	0.692
C-peptide index, ΔC30/ΔG30 (ng/mL per mmol/L)	0.412	0.318
TGs (mmol/L)	0.757	0.001
TCs (mmol/L)	0.589	0.023
HDL (mmol/L)	0.451	0.192
LDL (mmol/L)	0.489	0.122
GPT (U/L)	0.324	0.503
GOT (U/L)	0.222	0.753
hsCRP (mg/dl)	0.434	0.227
TNF (ng/L)	0.258	0.672
IL6 (pg/mL)	0.661	0.003

BMI: body mass index; SBP: systolic blood pressure; DBP: diastolic blood pressure; FPG: fasting plasma glucose; HOMA-IR: homeostasis model assessment of insulin resistance; HDL-C: high-density lipoprotein cholesterol; LDL-C: low-density lipoprotein cholesterol; TC: total cholesterol; TG: triglycerides.

## Data Availability

The authors declare that the data supporting the findings of this study are available within the article or are available from the corresponding author upon reasonable request.

## References

[1] Abella V., Pino J., Scotece M. (2017). Progranulin as a biomarker and potential therapeutic agent. *Drug Discovery Today*.

[2] Reinehr T. (2018). Long-term effects of adolescent obesity: time to act. *Nature Reviews Endocrinology*.

[3] Brady T. M. (2017). Obesity-related hypertension in children. *Frontiers in Pediatrics*.

[4] Nakamura K., Fuster J. J., Walsh K. (2014). Adipokines: a link between obesity and cardiovascular disease. *Journal of Cardiology*.

[5] Ouchi N., Ohashi K., Shibata R., Murohara T. (2012). Adipocytokines and obesity-linked disorders. *Nagoya Journal of Medical Science*.

[6] Daniel R., Daniels E., He Z., Bateman A. (2003). Progranulin (acrogranin/PC cell-derived growth factor/granulin-epithelin precursor) is expressed in the placenta, epidermis, microvasculature, and brain during murine development. *Developmental Dynamics*.

[7] He Z., Bateman A. (2003). Progranulin (granulin-epithelin precursor, PC-cell-derived growth factor, acrogranin) mediates tissue repair and tumorigenesis. *Journal of Molecular Medicine*.

[8] Qu H., Deng H., Hu Z. (2013). Plasma progranulin concentrations are increased in patients with type 2 diabetes and obesity and correlated with insulin resistance. *Mediators of Inflammation*.

[9] Hossein-Nezhad A., Mirzaei K., Ansar H., Emam-Gholipour S., Tootee A., Keshavarz S. A. (2012). Obesity, inflammation and resting energy expenditure: possible mechanism of progranulin in this pathway. *Minerva Endocrinologica*.

[10] Matsubara T., Mita A., Minami K. (2012). PGRN is a key adipokine mediating high fat diet-induced insulin resistance and obesity through IL-6 in adipose tissue. *Cell Metabolism*.

[11] Tonjes A., Fasshauer M., Kratzsch J., Stumvoll M., Bluher M. (2010). Adipokine pattern in subjects with impaired fasting glucose and impaired glucose tolerance in comparison to normal glucose tolerance and diabetes. *PLoS One*.

[12] Richter J., Focke D., Ebert T. (2013). Serum levels of the adipokine progranulin depend on renal function. *Diabetes Care*.

[13] Youn B. S., Bang S. I., Kloting N. (2009). Serum progranulin concentrations may be associated with macrophage infiltration into omental adipose tissue. *Diabetes*.

[14] Kloting N., Fasshauer M., Dietrich A. (2010). Insulin-sensitive obesity. *American Journal of Physiology. Endocrinology and Metabolism*.

[15] Tanner J. M. (1981). Growth and maturation during adolescence. *Nutrition Reviews*.

[16] Libman I. M., Barinas-Mitchell E., Bartucci A., Robertson R., Arslanian S. (2008). Reproducibility of the oral glucose tolerance test in overweight children. *The Journal of Clinical Endocrinology and Metabolism*.

[17] Ten S., Maclaren N. (2004). Insulin resistance syndrome in children. *The Journal of Clinical Endocrinology and Metabolism*.

[18] Valerio G., Licenziati M. R., Iannuzzi A. (2006). Insulin resistance and impaired glucose tolerance in obese children and adolescents from Southern Italy. *Nutrition, Metabolism, and Cardiovascular Diseases*.

[19] Bonora E., Targher G., Alberiche M. (2000). Homeostasis model assessment closely mirrors the glucose clamp technique in the assessment of insulin sensitivity: studies in subjects with various degrees of glucose tolerance and insulin sensitivity. *Diabetes Care*.

[20] Bacha F., Gungor N., Arslanian S. A. (2008). Measures of beta-cell function during the oral glucose tolerance test, liquid mixed-meal test, and hyperglycemic clamp test. *The Journal of Pediatrics*.

[21] Anakwe O. O., Gerton G. L. (1990). Acrosome biogenesis begins during meiosis: evidence from the synthesis and distribution of an acrosomal glycoprotein, acrogranin, during guinea pig spermatogenesis. *Biology of Reproduction*.

[22] Alissa E. M., Sutaih R. H., Kamfar H. Z., Alagha A. E., Marzouki Z. M. (2017). Serum progranulin levels in relation to insulin resistance in childhood obesity. *Journal of Pediatric Endocrinology & Metabolism*.

[23] Li H., Zhou B., Xu L. (2014). Circulating PGRN is significantly associated with systemic insulin sensitivity and autophagic activity in metabolic syndrome. *Endocrinology*.

[24] Smitka K., Maresova D. (2015). Adipose tissue as an endocrine organ: an update on pro-inflammatory and anti-inflammatory microenvironment. *Prague Medical Report*.

[25] Yin F., Banerjee R., Thomas B. (2010). Exaggerated inflammation, impaired host defense, and neuropathology in progranulin-deficient mice. *The Journal of Experimental Medicine*.

[26] Tanaka A., Tsukamoto H., Mitoma H. (2012). Serum progranulin levels are elevated in patients with systemic lupus erythematosus, reflecting disease activity. *Arthritis Research & Therapy*.

[27] Wei J., Hettinghouse A., Liu C. (2016). The role of progranulin in arthritis. *Annals of the New York Academy of Sciences*.

[28] Chitramuthu B. P., Bennett H. P. J., Bateman A. (2017). Progranulin: a new avenue towards the understanding and treatment of neurodegenerative disease. *Brain*.

[29] Liu F., Zhang W., Yang F. (2016). Interleukin-6-stimulated progranulin expression contributes to the malignancy of hepatocellular carcinoma cells by activating mTOR signaling. *Scientific Reports*.

[30] Weir G. C., Bonner-Weir S. (2004). Five stages of evolving beta-cell dysfunction during progression to diabetes. *Diabetes*.

[31] Nicoletto B. B., Canani L. H. (2015). The role of progranulin in diabetes and kidney disease. *Diabetology and Metabolic Syndrome*.

[32] Xu L., Zhou B., Li H. (2015). Serum levels of progranulin are closely associated with microvascular complication in type 2 diabetes. *Disease Markers*.

[33] Mozos I., Malainer C., Horbanczuk J. (2017). Inflammatory markers for arterial stiffness in cardiovascular diseases. *Frontiers in Immunology*.

